# Mapping the terrain: a scoping review of empirical studies on the big five personality traits and QoL in China

**DOI:** 10.3389/fpsyg.2023.1335657

**Published:** 2024-01-12

**Authors:** Cuiren Chen

**Affiliations:** School of Marxism, Dongguan Polytechnic, Dongguan, Guangdong, China

**Keywords:** personality traits, big five personality traits, openness, conscientiousness, extraversion, agreeableness, neuroticism, QoL

## Abstract

The relationship between personality traits and Quality of Life (QoL) has garnered increasing scholarly attention, particularly within the context of China. This scoping review synthesizes existing literature on the connection between the Big Five personality traits (Openness, Conscientiousness, Extraversion, Agreeableness, and Neuroticism) and QoL among the Chinese population. The review explores correlations, measurement instruments, and theoretical frameworks employed in these studies. The study’s significance lies in the profound implications for healthcare policies, organizational behavior, and social welfare programs in China, where rapid social and economic changes impact well-being. Investigating personality traits’ impact on QoL can inform tailored interventions benefiting diverse Chinese subpopulations. This review addresses three primary research questions: (1) How do the Big Five personality traits correlate with QoL in different Chinese subpopulations, and what are the mediating or moderating factors? (2) What instruments assess these traits and QoL in the Chinese cultural context, and how are they validated? (3) What theoretical or disciplinary frameworks guide this research in China? Following a systematic PRISMA-ScR framework and a customized “C-BFQLC” protocol, the study identified 170 relevant documents. After rigorous screening, ten studies met the inclusion criteria, constituting 5.03% of the initial records. This scoping review critically examines the interplay between the Big Five personality traits and QoL in China, a context marked by rapid socioeconomic changes and cultural diversity. Employing a systematic approach guided by the PRISMA-ScR framework and our unique ‘C-BFQLC’ protocol, we meticulously analyzed 170 documents, selecting nine that met our rigorous inclusion criteria. Despite the emerging nature of this research area in the Chinese context, our study reveals significant insights into how individual personality traits influence various dimensions of well-being. The implications of these findings are profound, extending beyond academic discourse to inform healthcare policies, organizational behavior, and social welfare programs in China. Our review not only offers a comprehensive synthesis of the current research landscape but also identifies critical gaps in the literature. It emphasizes the urgent need for further culturally nuanced research to understand the complex dynamics of personality traits and QoL in China. This work lays a foundational framework for future investigations and the development of tailored interventions aimed at enhancing the well-being of diverse Chinese populations, including the elderly, people with disabilities, and specific occupational groups.

## Introduction

Quality of Life (QoL) is a pivotal indicator of individual well-being and societal advancement, garnering attention from diverse stakeholders including the general public, social scientists, and policymakers. This multifaceted construct, which spans physical and mental health, social connectedness, job satisfaction, and personal growth, presents a complex and nuanced subject for study. Its broad spectrum and the myriad factors influencing it pose a significant challenge in isolating the impact of individual variables (Zhang et al., 2022). While existing literature often concentrates on specific dimensions of QoL or particular demographic groups, such as individuals with certain medical conditions (Pugi et al., 2022; Silva et al., 2022) or those experiencing life-changing events (Dwan and Ownsworth, 2019), a comprehensive exploration of how personality traits as a whole affect general QoL is notably lacking. This gap highlights the need for more expansive research to understand the overarching influence of personality traits on the various aspects of QoL, which could offer crucial insights for targeted policy and intervention strategies.

The Big Five personality traits—Openness, Conscientiousness, Extraversion, Agreeableness, and Neuroticism (OCEAN)—constitute a robust framework for understanding human personality (Costa and McCrae, 2000). Previous research has shown that these traits can exert a significant impact on diverse life outcomes, including mental health, occupational success, and social relationships. This makes it crucial to investigate the correlation between these traits and QoL, a construct that is multidimensional and encompasses physical, emotional, and social domains.

In recent years, the study of QoL has gained particular prominence in the context of China, a nation undergoing rapid social and economic transformations that are affecting both individual and collective well-being (Shi et al., 2022; Liu et al., 2023). Understanding these correlations has substantive implications for healthcare policies, organizational behavior, and social welfare programs within the country. A detailed examination of the personality traits that either positively or negatively correlate with QoL could inform targeted interventions aimed at elevating the well-being of diverse Chinese demographic groups, including the elderly, people with disabilities, and specialized occupational categories like judges and pilots.

This scoping review sets out to aggregate existing literature that explores the relationship between the Big Five personality traits and QoL, with a specific emphasis on studies carried out within the Chinese population. A scoping review of empirical studies is a systematic form of literature review that aims to map the existing body of research on a specific topic, identifying the range, nature, and extent of empirical studies conducted in the field, to clarify key concepts, and to pinpoint research gaps and emerging trends (Pollock et al., 2023). The review will focus on identifying the correlates, measurement instruments, and theoretical frameworks that are commonly employed in these studies. Three research questions directed at the Chinese population will be addressed:

a) Firstly, how do the Big Five personality traits correlate with QoL among various Chinese subpopulations, and what are the intervening or moderating factors influencing these relationships?
b) Secondly, what instruments are frequently used for assessing the Big Five traits and QoL, and how have these tools been validated in the Chinese cultural milieu?
c) Lastly, what theoretical or disciplinary frameworks are commonly referenced in this specialized field of research in China?

By concentrating on studies performed within the Chinese populace, this scoping review aims to furnish a comprehensive snapshot of the current state of research, identify lacunae in the existing body of literature, and propose avenues for future scholarly inquiry.

### Theoretical framework

#### Quality-of-life research

Quality of Life (QoL) is a broad, multi-dimensional construct that encompasses an individual’s overall well-being and life satisfaction, extending beyond mere health aspects. According to Ferrans and Powers (1985), QoL includes various facets of an individual’s existence, reflecting their perception of their position in life within the cultural and value systems they inhabit, and in relation to their personal goals, expectations, standards, and concerns. This definition, as outlined by the WHOQOL group in 1998, emphasizes the subjective evaluation of both positive and negative aspects of life. It’s important to distinguish QoL from Health-related Quality of Life (HQoL), which is more specifically concerned with how health status, including illness and treatment, impacts an individual’s quality of life. HQoL is a subset of the broader QoL concept, focusing particularly on health dimensions, whereas QoL, as defined here, encompasses a wider range of life domains, including social, emotional, and physical aspects (Whoqol, 1998). The dimensions of QoL usually encompass physical health, psychological state, level of independence, social relationships, and environmental factors (Park and Park, 2023; Puce et al., 2023).

In assessing QoL, a range of methodologies is employed, most commonly self-report questionnaires, interviews, and standardized tests. These assessment tools are validated through rigorous research and can be adapted for specific cultural and social nuances. The subjective nature of QoL makes self-report questionnaires especially pertinent, as they allow individuals to express their personal perspectives on their well-being (Liu et al., 2023).

The exploration of QoL holds considerable relevance in psychosocial research (Xie et al., 2023). Insights into how personality traits, such as those identified in the Big Five model, influence QoL can provide crucial intervention points for enhancing life satisfaction and general well-being (McCrae and Costa, 2003). For example, lower levels of neuroticism and higher levels of conscientiousness are commonly associated with better perceived QoL (Diener et al., 1999). These insights are particularly useful in therapeutic contexts and can also inform social policies and healthcare strategies aimed at enhancing community and societal well-being.

In summary, the multi-dimensional construct of QoL serves as an ideal platform for investigating how personality traits can impact diverse aspects of human life, thus enriching our comprehensive understanding of well-being (Haehner et al., 2023; Kaufman, 2023).

#### The Big five personality model

Personality refers to an individual’s enduring patterns of thought, emotion, and behavior that distinguish them from others (Allport, 1961). These patterns act as the psychological framework which remain consistent over various situations and over time (Sinclair et al., 2020). Within this broad construct, personality traits are the stable attributes that signify an individual’s consistent tendencies and behavioral preferences (Haehner et al., 2023).

The Big Five Personality Model, also known as the Five-Factor Model, has garnered empirical support as a comprehensive framework for understanding personality (John et al., 2008). Developed through lexical factor analyses, the model asserts that most individual differences in human personality can be classified under five broad dimensions: Openness, Conscientiousness, Extraversion, Agreeableness, and Neuroticism. Each dimension encapsulates a range of traits that define specific aspects of personality, such as imagination and originality for Openness or organization and dependability for Conscientiousness.

The Big Five model has been the subject of a plethora of studies, each substantiating its applicability across different cultures and practical realms like occupational psychology and education (Lucas and Diener, 2015; Martin, 2020). Seminal work by researchers such as Costa and McCrae have provided empirical foundations for the model. Nevertheless, critics argue that the model might be reductionist in nature and overlook culturally specific traits (Moreira et al., 2023).

The Big Five Personality Model, a cornerstone in psychosocial research, is particularly pertinent to our review as it provides a comprehensive framework for examining how personality traits intersect with various aspects of Quality of Life (QoL). This model enables a structured exploration of the correlations between personality traits—Openness, Conscientiousness, Extraversion, Agreeableness, and Neuroticism—and key social determinants such as education, socio-economic status, and health (Roberts et al., 2007). These determinants are integral to understanding the broader social context within which QoL is experienced and evaluated. Furthermore, the applicability of the Big Five Model in clinical settings, as noted by Roberts et al. (2009), is crucial for assessing psychological well-being, an essential component of QoL. By incorporating this model, our review aims to elucidate the nuanced ways in which individual personality differences interact with and impact the social and psychological dimensions of QoL. This intersectional approach allows for a more holistic understanding of QoL, highlighting the need for tailored interventions that address both psychological and social factors in promoting individual and societal well-being.

In conclusion, the Big Five Personality Model continues to be a pivotal framework for the academic and clinical study of personality, offering key insights into the relationship between individual differences and various elements within the psychosocial landscape.

#### The Big five personality model and its relationships with QoL

The relationship between personality traits, as described by the Big Five model, and QoL has garnered increasing attention in the academic literature. QoL is a multi-dimensional construct that encapsulates an individual’s physical, emotional, and social well-being (Fayers and Machin, 2013). The Big Five traits—Openness, Conscientiousness, Extraversion, Agreeableness, and Neuroticism—have been posited to play a considerable role in influencing various aspects of an individual’s QoL (Goodwin and Friedman, 2006).

The mechanisms through which these traits exert their influence are manifold. For instance, higher levels of Conscientiousness are often associated with better health behaviors, including regular exercise and a balanced diet, thereby positively influencing physical aspects of QoL (Bogg and Roberts, 2004). Extraversion, with its inherent sociability and positive emotionality, may enhance social and emotional aspects of QoL (Lucas and Diener, 2015). Conversely, high levels of Neuroticism have been linked to lower life satisfaction and well-being, often leading to diminished QoL (DeNeve and Cooper, 1998).

Several empirical studies have illustrated these relationships. For example, a study found that individuals with higher levels of Neuroticism were more likely to experience reduced QoL due to increased stress levels (Sutin et al., 2009). On the other hand, Goodwin, and Friedman showed that traits like Agreeableness and Conscientiousness were positively associated with self-reported life satisfaction, a key component of QoL (Goodwin and Friedman, 2006). Gale et al. further expanded on this by exploring how Openness was correlated with a willingness to engage in new experiences that could enhance one’s psychological well-being (Gale et al., 2013).

In summary, the Big Five Personality Model provides a comprehensive framework for understanding the nuanced ways in which individual personality traits can influence QoL. These influences manifest through various mechanisms, such as health behaviors, emotional stability, and social interactions. The body of empirical research supporting these associations underscores the importance of considering personality traits when evaluating or seeking to improve an individual’s QoL.

#### QoL and Big five personality model research in China

QoL studies in China have often been grounded in the nation’s unique socio-economic and cultural contexts (Huang et al., 2023). The rapid economic development in recent decades has led to profound changes in individual and societal well-being (Xu and Wu, 2022; Zhang et al., 2022). However, such gains have not always translated into improved QoL (Lo and Wang, 2018), particularly in terms of psychological well-being and social relationships (Wang et al., 2020). When it comes to the Big Five Personality Model, research in China has been significantly influenced by indigenous cultural perspectives, particularly Confucian values (Jiang and Wei, 2022). Traits like Agreeableness and Conscientiousness often find resonance with Confucian virtues such as ‘Ren’ (benevolence) and ‘Yi’ (righteousness) (Cheung and Leung, 1998). However, critics have pointed out that Western-based models like the Big Five may not wholly capture the complexities of Chinese personality structures (Chen and Chen, 2004), necessitating the incorporation of native traits such as “face” and “guanxi” (relationships) (Liu, 2021).

Recent empirical studies have started to blend Western and Chinese perspectives. In China, research on QoL and the Big Five Personality Model reflects an intricate interplay between global psychological frameworks and local cultural norms (Montag et al., 2022). The empirical evidence demonstrates the complexity of these relationships (Zhai et al., 2010), influenced as they are by economic, social, and cultural factors unique to China (Carleo, 2021).

The aim of this work is to conduct a comprehensive scoping review of empirical studies centered on the relationship between the Big Five Personality Traits and QoL in China. The objectives of this scoping review are manifold:

a) To chart the development and distribution of publications on the Big Five Personality Traits and QoL within the Chinese context, providing insights into the trajectory of this research area.
b) To outline the fundamental methodological characteristics employed in these studies, including research design, sample demographics, and measurement instruments, thus offering a critical evaluation of the existing body of work.
c) To execute a content analysis that categorizes the various correlates explored in association with the Big Five Personality Traits and QoL in China, and to ascertain the types of interventions reported, if any.

In doing so, the review seeks to provide a clear, objective understanding of the current research landscape in this area. It will examine how these personality traits correlate with different aspects of QoL in the Chinese setting, thereby offering insights into the unique interplay of psychological and sociocultural variables in shaping individual well-being. This approach serves not only to highlight the existing knowledge but also to pinpoint the areas requiring further investigation, setting the stage for future research initiatives in this field. Three research questions directed at the Chinese population will be addressed:

a) Firstly, how do the Big Five personality traits correlate with QoL among various Chinese subpopulations, and what are the intervening or moderating factors influencing these relationships?
b) Secondly, what instruments are frequently used for assessing the Big Five traits and QoL, and how have these tools been validated in the Chinese cultural milieu?
c) Lastly, what theoretical or disciplinary frameworks are commonly referenced in this specialized field of research in China?

## Method

As guided by the PRISMA-ScR framework and in line with the JBI Evidence Synthesis Manual (Aromataris, 2020), we have devised a protocol to ensure the consistent application of criteria across all stages of the research process. This extends from the preliminary search for academic articles to the final inclusion of papers specifically focusing on the relationship between the Big Five Personality Traits and QoL within the Chinese context. This structured approach aims to maintain rigor and uniformity in the methodology, thereby enhancing the validity and reliability of the scoping review’s findings as they pertain to the Big Five Personality Traits and QoL in China.

### Inclusion and exclusion criteria

In alignment with the research question and by adapting the PICO strategy for our specific focus, we formulated a customized protocol termed “C-BFQLC” (Concept, Big Five, QoL, Context). This methodology was developed in consultation with established guidelines and scholarly recommendations (Munn et al., 2018a,b), tailored specifically to explore the relationship between the Big Five Personality Traits and QoL in a Chinese setting. This specially designed protocol ensures a systematic approach to our inquiry, offering a structured framework for identifying, evaluating, and interpreting the available empirical data. The C-BFQLC protocol aims to provide a coherent and comprehensive understanding of how the Big Five Personality Traits intersect with Quality-of-Life outcomes within the sociocultural and economic landscape of China (Please, see Table 1).

**Table 1:** Inclusion and exclusion criteria for studies on big five personality traits and QoL in China.

Criteria	Inclusion	Exclusion
Concept (CBFQoL)	Studies specifically examining the Big Five personality traits Model and their impact on QoL	Studies not directly related to the Big Five or QoL
Context (Co)	Research conducted in China or includes Chinese population in the sample	Research conducted outside China without a Chinese population
Participants (p)	Chinese individuals aged 18 years and above	Non-Chinese individuals or those under 18 years
Publication Type	Peer-reviewed journal articles, systematic reviews, and meta-analyses	Magazine articles, editorials, conference abstracts, non-peer-reviewed reports
Assessment (a)	Validated scales measuring the Big Five personality traits and QoL, such as the NEO Five-Factor Inventory (NEO-FFI) or the Short Form Health Survey (SF-36)	Single-item scales or non-validated instruments for measuring the Big Five or QoL
Study Design (s)	Longitudinal, cross-sectional, and experimental studies	Case reports, qualitative studies, and theoretical reviews

### Concept

The primary focus of this review is on publications that assess the Big Five personality traits, using the Big Five Questionnaire (BFQ) or others, and its impact on QoL. This concentration ensures that the review captures studies that are directly relevant to understanding how individual differences in personality traits relate to Quality-of-Life outcomes within the Chinese context.

### Context

The scope of this review is specifically narrowed to publications that investigate the relationship between the Big Five personality traits, as measured by the Big Five Questionnaire (BFQ), and QoL within the Chinese context. Accordingly, studies that explore the Big Five personality traits in general or in contexts other than QoL, as well as those conducted outside China, have been excluded from this review.

### Participants

For this review, the participant criteria were set to include studies that focus on individuals aged 18 years and above within the Chinese context. This age group was selected for its relevance to fully understanding how personality traits, specifically the Big Five, influence QoL across different stages of adulthood. Studies that included minors, non-Chinese populations, or failed to specify the age or nationality of the participants were not considered for inclusion.

### Publication type

For this review, we included both periodical and non-periodical academic publications. Specifically, peer-reviewed journal articles, systematic reviews, and meta-analyses were considered for inclusion. These sources were chosen for their empirical rigor and academic credibility in studying the relationship between the Big Five personality traits and QoL within the Chinese context. In contrast, magazine articles, editorials, conference abstracts, and other similar types of non-peer-reviewed documents were excluded from the study. The exclusion of these sources was based on the need for empirical rigor and validated methodologies to ensure the academic integrity of the review.

### Mode of assessment and sample size

In this review, we included empirical studies that explicitly stated both the sample size and the mode of assessment for the Big Five personality traits. Studies were considered for inclusion if the Big Five were assessed using validated multi-item scales, such as the Big Five Inventory (BFI) or the NEO Five-Factor Inventory (NEO-FFI), and if these measures were used to examine their impact on QoL through validated scales like the Short Form Health Survey (SF-36), or the World Health Organization QoL Abbreviated Version (WHOQOL-BREF). On the other hand, studies that used single-item instruments or unstructured, non-validated scales to measure either the Big Five traits or QoL were excluded from the review.

### Study design

In this review, we specifically included empirical studies that investigate the relationship between the Big Five personality traits and QoL. These studies were chosen for their ability to provide objective data and insights into the causal or correlational links between personality traits and QoL outcomes. Excluded from consideration were theoretical studies, literature reviews, and case studies, as they do not offer the empirical evidence required to substantiate the relationships under examination.

### Search strategy

The search equation was developed by the author and refined in consultation with experts in psychometric assessment and health-related QoL studies. Boolean operators (AND & OR) and truncation techniques (* and inverted commas) were applied to fields such as Title (TI), Abstract (AB), and Keywords (KW). The final search strategy was constructed as follows: [(“Big Five” OR “Five Factor Model” OR “NEO-FFI” OR “BFI”) AND (“QoL” OR “Well-being” OR “Life Satisfaction”)] AND [“China” OR “Chinese population”]. This search was performed to include publications up to September 2023.

In databases like ProQuest, a Natural Language Full Text search (NOFT) was utilized: [(“Big Five” OR “Five Factor Model”) AND (“QoL” OR “Well-being”) AND “China”]. For the Web of Science (WoS) database, the search was conducted in the TOPIC field using the same search terms, and it included publications up to September 2023.

### Formal strategies

The literature for this review was sourced through a multi-step process to ensure comprehensive coverage of the topic. Initially, several automated databases were utilized for the search, specifically focusing on the fields of Psychology, Behavioral Sciences, and Health. These databases included ERIC, MedLine, Psychology and Behavioral Sciences Collection, PsycINFO, PubPsych, and Teacher Reference Center for thematic searches, as well as Academic Search Ultimate, E-Journals, ProQuest, Scopus, and Web of Science for multidisciplinary perspectives. The search was conducted without any language restrictions to maximize the inclusivity of relevant international research. Furthermore, the bibliographies of located articles were reviewed to identify any additional publications that might be of relevance to the study of the Big Five personality traits and QoL.

### Coding and identification of records and data extraction

A structured protocol was established to delineate the fields for data extraction and analysis. Bibliometric data points included: (a) year of publication; (b) authorship; (c) title; (d) source of publication, such as journal or book; (e) DOI; and (f) abstract. Additional enriched fields, structured in accordance with the C-BFQLC format, were added to include: (a) Document typology; (b) Concept, specifying whether the document focused on the Big Five and QoL; (c) Context, indicating if the study was specifically focused on the Chinese population; (d) Study design, noting if it was empirical, theoretical, or a review; (e) Participant details, including number, type, education level, and geographical origin; (f) Evaluation methods used for assessing the Big Five and QoL; (g) Type of research design, such as experimental or observational; (h) Type of intervention, if applicable; (i) Analyzed variables, categorized into various domains like sociodemographic, psychological well-being, and health; (j) Outcomes; and (k) Conclusions relevant to the Big Five and QoL.

Throughout the coding and data extraction process, regular meetings were organized to resolve any inconsistencies or disagreements. Each record was independently reviewed by the author and an independent expert, examining the title, abstract, and full text to ensure comprehensive coding. The subsequent phase involved two types of analyses: (a) a thematic content analysis of the variables, and (b) a classification of interventions, if applicable, based on the variables studied.

### Quality of the empirical studies included in the present systematic reviews

The assessment of the studies’ methodological rigor was conducted using a standardized tool designed for evaluating quantitative research (Sirriyeh et al., 2012). This assessment focused on a range of criteria to determine each study’s appropriateness and thoroughness. The criteria included in our database encompassed several key aspects: (a) the presence of a well-defined theoretical framework; (b) clear articulation of aims and objectives within the report; (c) detailed description of the research context; (d) consideration of sample size in relation to the analytical approach; (e) representation and size of the sample relative to the target population; (f) comprehensive outline of the data collection methods; (g) evaluation of the measurement tools’ reliability and validity through statistical methods; (i) robust rationale for the chosen analytical technique; (j) critical examination of the study’s strengths and limitations. Each paper was then assigned a Global Quality Score, ranging from (3) indicating strong methodology, to (0) denoting weak methodology.

## Results

### Screening process

**Figure 1:**
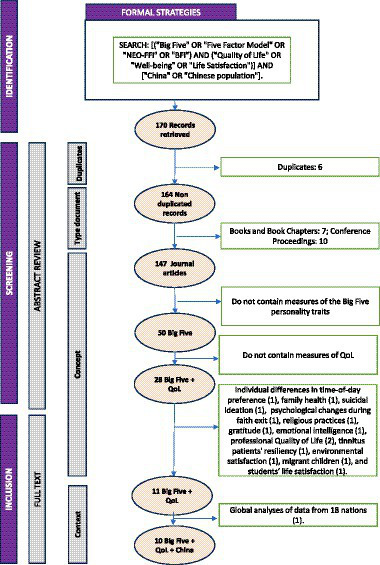
Fflowcharts of the review process.

The implemented search strategies resulted in the identification of 170 documents pertinent to the Big Five personality traits and QoL. In the first step, six studies have been identified as duplicates. The remaining 164 records have been retrieved and carefully revised by content analyses within the title and the abstract. Seventeen books, chapters and Conference proceedings were eliminated. In the first screening, 97 studies have been excluded because they do not contain measures of the Big Five personality traits. Among them, 11 studies were focused on medical topics as “cancer,” “breast,” “depression,” and “obesity.” A second group of 22 studies were focused on social aspects of age groups (“older,” “adults”). A third group of 9 studies likely addresses environmental concerns, such as “energy,” “coastal,” “conservation,” and “environmental.” A fourth group of 17 studies were focused on medical interventions and risk factors, as “stroke,” and others. A group of 11 studies discussed professional life, and 16 focused on urban and spatial development. Finally, a total of 11 studies seem to delve into psychological aspects of media usage. From the remaining studies, 21 have been excluded due to the absence of content related to QoL, and 29 studies were retained for full text examination, in order to be included in the review concerning these topics in China. One study has been excluded for global analyses of data from 18 nations (Bond et al., 2020), additional studies have been excluded due to its focus on other topics, as individual differences in time of day preference (Carciofo et al., 2016). family health (Hao S. et al., 2023), suicidal ideation (Huang et al., 2019), psychological changes during faith exit (Hui et al., 2018), religious practices (Hui et al., 2011), gratitude (Kong et al., 2020), emotional intelligence (Law et al., 2004), professional QoL (Yu et al., 2016; Li and Xie, 2020), tinnitus patients’ resiliency (Xin et al., 2022). environmental satisfaction (Zhai et al., 2010), migrant children (Zhang, 2018) and students (Xu et al., 2017). Finally, only 10 met the established inclusion criteria, accounting for 5.03% of the initial records identified (Feng et al., 2014; Zhang et al., 2015; Kong et al., 2020; Li Z. M. et al., 2020; Tian et al., 2020; Lai and Li, 2022; Li T. et al., 2022; Lin et al., 2022; Yu et al., 2022; Cai et al., 2023). Figure 1 illustrates the comprehensive process undertaken, detailing the selection of formal and informal strategies used for sourcing literature. Additionally, it provides a breakdown of the number of records at each stage, along with the specific reasons for the inclusion or exclusion of each document during the various phases of the screening process.

The earliest work that empirically treated the relationships between BF and QoL was published in 2014. Since then, the evolution of publications in this specific area has been irregular over the last nine years, indicating fluctuating scholarly interest in the topic.

These sections offer a transparent look at the literature search and selection process, as well as a historical overview of the field, thereby enhancing the rigor and validity of this review on the Big Five personality traits and QoL.

### Characteristics of the studies

The characteristics of the included studies are presented below: Participants, instruments for assessment of main variables, and type of design.

#### Content analyses

Table 2 contains the characteristics of the 10 included studies. The studies under review primarily investigate the relationship between the Big Five personality traits and QoL in various populations and settings. A common thread across these studies is the focus on understanding how individual personality traits are correlated with different aspects of QoL, often employing advanced statistical methods like structural equation modeling for this purpose.

**Table 2:** Studies characteristics.

Type of document	Authors (Year)	Participants		Main findings	QGR
		Sample size	Type of participants		
A	Cai et al. (2023)	*N* = 358; 193 women and 165 men.	People with Disabilities	QoL is positively associated with four out of the five Big Five personality traits—specifically, extraversion, agreeableness, conscientiousness, and openness. Neuroticism was negatively correlated with QoL Social support significantly mediated the positive relationship between extraversion, agreeableness, conscientiousness, openness, and QoL, it did not have a mediating effect on the relationship between neuroticism and QoL.	++
A	Feng et al. (2014)	*N* = 330	Judges undergoing professional training at the Shandong judge training institute.	Substantial negative direct effect of neuroticism on QoL, as well as a negative indirect effect mediated through perceived occupational stressors. Specifically, neuroticism not only directly diminished QoL but also increased the perception of occupational stressors, which in turn had a further detrimental effect on QoL. In contrast, the other Big Five personality traits—openness, conscientiousness, agreeableness, and extraversion—did not show significant direct or indirect effects on QoL when mediated by perceived occupational stressors.	+++
A	Kong et al. (2020)	*N* = 559	Civil servants aged between 27 to 60 years from Shandong province in China.	Neuroticism is shown to have both direct and indirect negative effects on QoL. Specifically, neuroticism directly lowers QoL and also has an indirect negative impact through its positive correlation with occupational stress and negative correlation with job satisfaction. Furthermore, occupational stress itself directly lowers QoL and has an additional indirect negative effect mediated through its impact on job satisfaction.	++
A	Lai and Li (2022)	*N* = 253	Hong Kong Chinese older adults aged 60 and above.	Significant association between neuroticism and QoL, while no such relationship was observed for extraversion. Resilience serves as a mediating factor in the relationship between neuroticism and QoL. Interestingly, the mediating effect of resilience is more pronounced among participants who are in financially poor or fair conditions compared to their wealthier counterparts.	++
A	Li Y. et al. (2022)	*N* = 1,147	Older adults from a cross-sectional survey across more than 120 cities in China.	During the COVID-19 pandemic, nearly half of the elderly population experienced mild to severe levels of depression. Factors such as physical health, extraversion, conscientiousness, agreeableness, and family support were found to be negatively correlated with depression levels. Conversely, neuroticism and media use were positively associated with elevated levels of depression among the elderly.	+++
A	Li Z. M. et al. (2020)	*N* = 642	Patients with type 2 diabetes mellitus from 4 community healthcare services and 22 affiliated community stations in Beijing	Neuroticism was found to be positively correlated with perceived disorders in medication and exercise. On the other hand, conscientiousness, agreeableness, and extraversion were positively associated with perceived benefits of exercise. Additionally, neuroticism was positively correlated with perceived disorders in diet, while conscientiousness and agreeableness were negatively correlated with the same.	++
A	Lin et al. (2022)	*N* = 77 participants and *N* = 32 age and gender-matched controls.	Participants receiving orthognathic surgery, and age and gender-matched controls	Before orthognathic surgery, findings showed the extraversion had significant difference between the male and female orthognathic surgery subgroups.	++
A	Tian et al. (2020)	*N* = 286	Elder adults who were randomly recruited from among cycling participants in China.	Significant positive relationship between personality and cycling specialization, both of which were positively correlated with subjective well-being among older adults. Notably, personality had an indirect, positive effect on subjective well-being, mediated through cycling specialization as well as cognitive and affective factors.	++
A	Yu et al. (2022)	*N* = 220	Male pilots with an average age of 33.31 years.	The mediating effect of personality factors between resilience and the QoL of pilots was observed. Personality factors also mediated the relationship between social support and the mental health of pilots.	++
A	Zhang et al. (2015)	*N* = 2,270 depressive group (*n* = 474) and non-depressive group (*n* = 1,416)	Undergraduate college students. Mean Age = 20 years.	Undergraduates present a relatively high level of assessment of life quality. Neuroticism may be associated with depressive status and subjective assessment of life quality. Subjects with high neuroticism are susceptible to depressive status and poor subjective assessment of life quality.	+

Type of document: A (Journal Article). QGR, Quality Global Rating for this paper. +++, Strong; ++, Moderate; +, Weak.

a Investigated Correlates: The correlates frequently investigated include social support, resilience, occupational stress, and job satisfaction. These variables often serve as mediators or moderators in the relationship between BF and QoL. The set of investigated variables, as well as their correlates, moderators and mediators are detailed as follows. The Big Five traits—Extraversion, Agreeableness, Conscientiousness, and Openness—are shown to have a positive correlation with QoL. These positive effects are further enhanced by social support, represented as a mediator. In contrast, Neuroticism negatively correlates with QoL and its effects are amplified by occupational stress. Resilience is a nuanced mediator that especially comes into play in individuals experiencing financial hardship. Situational factors like the COVID-19 pandemic are also included, highlighting their impact on levels of depression among the elderly. Health behaviors and special contexts like cycling and aviation show how personality traits can influence various aspects of well-being and mental health.
b) Area of Studies: The studies span multiple domains, including psychology, healthcare, and occupational research. They cover diverse populations such as people with disabilities, civil servants, judges, pilots, and older adults.
c) Behaviors or Topics Included: Several studies delve into specific behaviors or health outcomes, such as medication and exercise attitudes in patients with type 2 diabetes, mental health conditions like depression among the elderly, and occupational stress among judges. Some studies also explore the impact of life-altering interventions like orthognathic surgery on psychological well-being and QoL.

**Figure 2:**
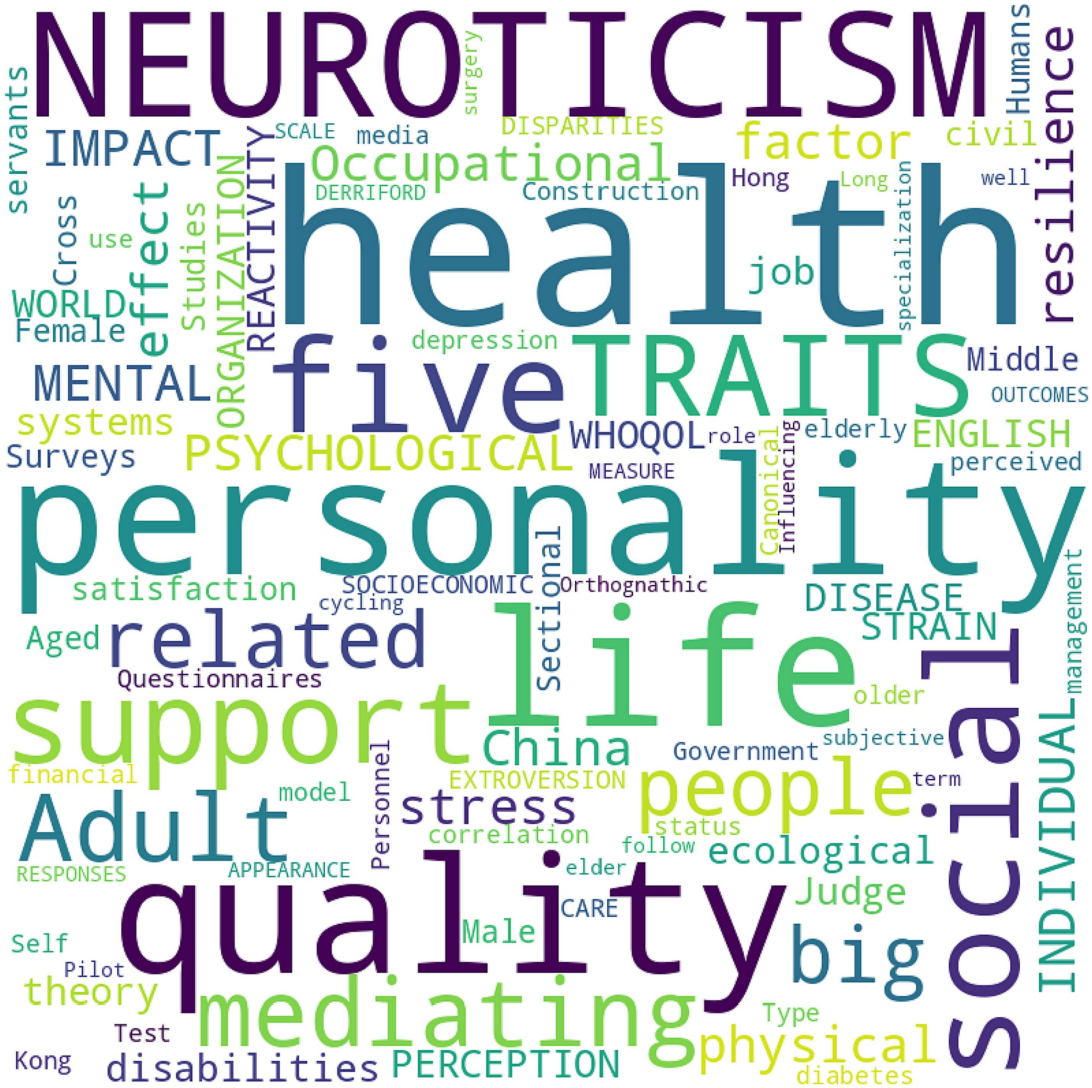
Word cloud created from keywords of the included articles based on the relevant terms occurring, and the size of each word in the word cloud corresponds to its frequency in the dataset.

Based on the articles’ keywords, the objectives, main topics and psychological aspects specifically tackled in the papers included in this review are represented in Figure 2. The word cloud provides a visual representation of the frequency of keywords across a range of articles. In this specific word cloud, several terms stand out due to their larger font size, indicating their prominence or frequency in the dataset. *QoL* is noticeably larger than most others, signifying its frequent occurrence. This suggests that QoL is a central theme in the articles from which these keywords are derived. *Neuroticism* and *Personality*: These terms are also quite prominent, indicating that psychological traits and their impact on various life outcomes are significant areas of focus. Neuroticism, one of the Big Five personality traits, seems to be of particular interest. *Social Support*: This term, while not as large as some others, still stands out. This may imply that the role of social networks and interpersonal relationships is considered important in the studied contexts. *China*: The prominence of this geographical keyword could indicate a regional focus in the articles, possibly examining social, psychological, or health-related phenomena specifically within the Chinese context. *Physical Health* and *Mental Health*: Both terms appear in the word cloud, indicating a balanced focus on various aspects of health, not just psychological or emotional factors. *Traits*, *Adult*, *Individuals*: These terms appear more than once, suggesting that the articles may often focus on individual-level analyses and may span across different age groups. *Mediating Effect*: This term suggests that some of the articles are likely investigating intermediary variables that influence the relationship between different primary variables.

d) The studies in the review predominantly draw from the disciplines of psychology, healthcare, and occupational research. Psychological frameworks, particularly those rooted in personality psychology, serve as the foundation for exploring the Big Five personality traits and their impact on QoL. The healthcare-oriented studies often incorporate frameworks from health psychology, focusing on health-related QoL, and may employ medical models to assess well-being in specific patient populations. Occupational studies typically engage with organizational psychology, examining how personality traits influence job-related stress and satisfaction, which in turn affect QoL. Overall, the studies operate at the intersection of these disciplines, applying their respective theoretical frameworks to offer a nuanced understanding of the complex relationships between personality traits and various aspects of QoL.

a) Participants

The total number of participants across all the studies is 6,342. On average, each study includes approximately 430 participants, with a standard deviation of approximately 300.3. This suggests that while the average number of participants per study is around 430, there is significant variability in the sample sizes across the different studies. The participants included in the articles of this systematic review on the Big Five personality traits and QoL (QoL) are quite diverse, encompassing various demographic and occupational groups. Below is a description of the types of participants: People with Disabilities, Chinese Judges; Civil Servants; Hong Kong Chinese Older Adults; Older Adults During COVID-19; Patients with Type 2 Diabetes Mellitus; Orthognathic Surgery Patients; Elderly Cyclists; adult college students, and Male Pilots.

b) Assessment of the main variables

Various instruments have been used to assess the Big Five personality traits and QoL. Below is a summary of the types and characteristics of these instruments, displayed in Table 3.

**Table 3:** Instruments for assessing the main variables.

*Instruments for Assessing Big Five Personality Traits*
Big Five Inventory (BFI): Used in multiple studies, BFI is a standard questionnaire designed to measure the high-order personality traits known as the Big Five.
Chinese Big Five Personality Inventory: Specifically employed in the study involving patients with type 2 diabetes mellitus, this variant is likely adapted for the Chinese population.
Big Five Personality Inventories: Mentioned in the study on pilots, although it’s unclear if this refers to BFI or another established measure.
*Instruments for Assessing QoL*
General Questionnaire: In the first study on people with disabilities, a general questionnaire was used to measure QoL variables, although the exact name of the instrument is not mentioned.
Medical Outcomes Study Short-Form Health Survey (SF-36): Used in the study on Chinese judges, SF-36 is a widely used health survey for assessing various dimensions of health-related QoL.
Short Form 8 (SF-8): Employed in the study on Hong Kong Chinese older adults, SF-8 is a shorter version of SF-36 and is used to assess health-related QoL.
36-Item Short-Form-Health-Survey (SF-36): Used in the study on orthognathic surgery patients, similar to the one used for Chinese judges.
World Health Organization QoL Abbreviated Version (WHOQOL-BREF): used in the Zhang study, it is a self-report questionnaire that contains 26 items and addresses 4 QOL domains: physical health (7 items), psychological health (6 items), social relationships (3 items) and environment (8 items), with the remaining two items measuring overall QOL and general health.
Symptom Checklist-90: Used in the study on pilots, this instrument is more aligned with mental health but could be considered as an indirect measure of QoL.
*Instruments for Assessing Mediating or Related Variables*
Social Support Indicators: Used in the study on people with disabilities, these appear to mediate the relationship between BF and QoL.
Occupational Role Questionnaire (ORQ): Used in the study on Chinese judges to measure perceived occupational stressors.
Connor-Davidson Resilience Scale (CD-RISC): Employed in multiple studies, this scale measures resilience, which appears to mediate the relationship between BF and QoL.

The instruments utilized for measuring the Big Five personality traits and QoL are generally well-established scales, but they are not specifically tailored for any unique subpopulations like people with disability or elderly. Rather, these instruments, such as the Big Five Inventory (BFI) and the Medical Outcomes Study Short-Form Health Survey (SF-36), are often validated in adult populations and then applied more broadly. Notably, the most frequently used instrument for assessing personality appears to be the Big Five Inventory, while the Medical Outcomes Study Short-Form Health Survey (SF-36) is commonly employed for evaluating QoL. These instruments are typically used in their entirety, capturing multiple dimensions of personality and QoL, rather than selective portions of the scales.

c) Study design

The studies included in the review predominantly employ cross-sectional designs, which serve to capture a snapshot of the relationships between the Big Five personality traits and QoL at a single point in time. It’s worth noting that several studies also employed structural equation modeling to test the relationships between variables. While cross-sectional studies are valuable for identifying correlations, they are limited in establishing causality. It is also noteworthy that some of the studies focus on specific populations, such as judges, pilots, and people with disabilities, and undergraduate college students, which may limit the generalizability of the findings but offer in-depth insights into these particular groups. Overall, the cross-sectional design appears to be the favored approach for examining the intricate relationships between personality traits and QoL, although the inclusion of longitudinal studies in future reviews could enrich our understanding of how these relationships evolve over time.

Quality assessment of empirical studies showed that only 2 pieces of research reached the strongest values using the tool for quantitative studies. Reduced samples, with only a cross-sectional design, convenience sampling procedures and lack of adequate justification of the participants’ inclusion and the analytical procedures were the main reasons for medium and lower values of Quality Global Rating, as displayed in Table 2.

## Discussion

The primary objective of this scoping review was to meticulously explore the empirical research conducted in China, focusing on the Big Five personality traits and their correlation with Quality of Life (QoL). This research holds significant importance as it sheds light on the intricate ways in which personality, as conceptualized by the Big Five model, influences various aspects of QoL in a culturally rich and dynamically evolving Chinese context. By examining these relationships within China’s unique socio-cultural milieu, this study contributes to the broader understanding of personality traits’ universality and cultural specificity. Importantly, it addresses the critical issue of cross-cultural invariance of the Big Five personality traits, a topic of ongoing debate in psychological research. Our findings not only reinforce the relevance of these personality dimensions across different cultures but also highlight the nuanced ways in which cultural contexts can shape and interact with these traits to influence individual well-being.

First Research question: *how do the Big Five personality traits correlate with QoL in various Chinese subpopulations, and what are the mediating or moderating factors in these relationships?*

In the context of Chinese subpopulations, the Big Five personality traits exhibit varied correlations with QoL. Extraversion, agreeableness, conscientiousness, and openness generally have a positive association with QoL. In contrast, neuroticism consistently shows a negative correlation. Zhang et al. (2015) study comparison between depressive and non-depressive undergraduate college students showed that neuroticism was negatively associated with the overall and the four domains of WHOQOL-BREF. The role of mediating and moderating factors adds complexity to these relationships.

Social support emerges as a significant mediator, particularly in enhancing the positive effects of extraversion, agreeableness, conscientiousness, and openness on QoL. However, it does not mediate the negative relationship between neuroticism and QoL. Occupational stress and job satisfaction serve as additional mediators, particularly amplifying the negative effects of neuroticism on QoL. The impact of these occupational factors also suggests that context-specific stressors can modulate the personality-QoL relationship.

Resilience plays a nuanced mediating role, particularly in the relationship between neuroticism and QoL. Its effect is more pronounced in individuals experiencing financial hardship, indicating that socio-economic factors can serve as moderators. During the COVID-19 pandemic, various personality traits were found to correlate with levels of depression among the elderly, highlighting the role of situational factors in affecting QoL.

Health behaviors and attitudes toward medication, exercise, and diet also reveal correlations with the Big Five traits, with neuroticism often linked to poorer health behaviors and attitudes. In specialized contexts such as cycling among older adults, personality traits not only influence the activity (cycling specialization) but also have an indirect (Tian et al., 2020) positive impact on subjective well-being, mediated through the activity and cognitive and affective factors.

In aviation, personality factors mediate the relationship between resilience, social support, and the mental health of pilots, indicating the applicability of these findings across occupational groups.

Overall, the relationship between the Big Five personality traits and QoL in Chinese subpopulations is multifaceted, influenced by a range of mediating and moderating factors, including social support, occupational stressors, resilience, and specific life circumstances.

Second Research question*: what types of instruments are commonly used to assess BF and QoL, and how are these instruments validated within the Chinese cultural context?*

In the reviewed studies conducted within Chinese subpopulations, various instruments are employed to assess the BF personality traits and QoL. For assessing the Big Five personality traits, the Big Five Inventory (BFI) is commonly used across multiple studies. A culturally specific variant, the Chinese Big Five Personality Inventory, was used in research involving patients with type 2 diabetes mellitus, indicating an adaptation for the Chinese cultural context (Li Z. M. et al., 2020). The term “Big Five Personality Inventories” also appeared in a study on pilots, although it was unclear whether this referred to BFI or another established measure.

Regarding Quality-of-Life assessment, several instruments are in use. A general questionnaire was utilized in the first study on people with disabilities, but its exact nature was not specified (Cai et al., 2023). The Medical Outcomes Study Short-Form Health Survey (SF-36) featured in studies on Chinese judges and orthognathic surgery patients. Its shorter version, the Short Form 8 (SF-8), was used in research on Hong Kong Chinese older adults. Both SF-36 and SF-8 are standard instruments for assessing health-related QoL. The Symptom Checklist-90 appeared in the study on pilots, primarily aligned with mental health but also serving as an indirect measure of QoL. The WHOQOL-BREF, that it is the short version of the WHOQOL 100 and is recommended for use when time is restricted or the burden on the respondent needs to be minimized. This survey has been used in large epidemiological studies and clinical trials, and also used by Zhang et al. (2015) study.

Instruments for assessing mediating or related variables include social support indicators, the Occupational Role Questionnaire (ORQ), and the Connor-Davidson Resilience Scale (CD-RISC). These instruments were used to measure factors like social support and occupational stressors, which have been shown to mediate the relationship between the Big Five personality traits and QoL.

While the studies do not explicitly detail the validation process of these instruments within the Chinese cultural context, the use of culturally adapted variants like the Chinese Big Five Personality Inventory suggests an awareness of the need for cultural adaptation. The diverse range of instruments reflects the multidimensional nature of both personality traits and QoL, as well as the complex interplay between them.

Related to this question is the concept of cross-cultural invariance of the Big Five personality traits, that it is a pivotal aspect in understanding their universality and applicability across diverse cultural contexts. The Big Five model, originally developed in Western settings, has been a subject of extensive research to determine its relevance and consistency in non-Western cultures, including China. Our review has contributed to this discourse by exploring how these personality dimensions’ manifest and influence Quality of Life (QoL) within the unique socio-cultural framework of China. The findings suggest that while the core components of the Big Five traits are identifiable across cultures, their expressions and impacts on QoL can be significantly modulated by cultural factors. For instance, the way Conscientiousness or Extraversion relates to well-being in the Chinese context may differ from Western interpretations, reflecting the deep influence of cultural norms, values, and societal expectations. This observation underlines the necessity of considering cultural nuances when applying the Big Five model in cross-cultural research, emphasizing that while personality traits may have a universal framework, their manifestations and implications are deeply rooted in cultural contexts. Such insights are crucial for developing more culturally sensitive psychological assessments and interventions, and for advancing our understanding of personality psychology in a globalized world.

Third Research question: *what theoretical or disciplinary frameworks are frequently invoked in this area of research within China?*

In the context of Chinese subpopulations, the studies reviewed suggest a complex interplay between Big Five personality traits and QoL, mediated by various factors such as social support, occupational stress, and resilience (Grevenstein et al., 2018). Neuroticism consistently shows a negative correlation with QoL across diverse groups including people with disabilities, civil servants, undergraduate college students, and judges. Conversely, traits like extraversion, agreeableness, conscientiousness, and openness generally have a positive impact on QoL, particularly when mediated by social support. However, the role of these traits becomes less clear in specific occupational settings. For instance, among judges and civil servants, neuroticism’s effect on QoL is further mediated by occupational stress, but the other Big Five traits do not exhibit the same pattern. The mediating role of resilience in the relationship between personality traits and QoL also emerges as significant, particularly among older adults in varying financial conditions (Lai and Li, 2022). Moreover, the influence of personality traits extends to specialized behaviors and attitudes, such as medication and exercise regimes in type 2 diabetes patients, and even cycling specialization among the elderly. In a similar vein, studies that include other disciplinary frameworks, as Psychophysiology (Kong et al., 2020), as well as different types of mediator variables among predictors and outcomes (Datu and Lin, 2022; Wang et al., 2022a,b), can expand our view of the topics.

Despite these insights, several gaps in the literature warrant further attention. For example, there’s a need for more nuanced investigations into how Big Five traits interact with occupational stressors (Zhou et al., 2017; Hao R. et al., 2023; Li et al., 2023), mainly in some specific professions (Wang et al., 2022a,b; Yang H. et al., 2022; Yang P. Y. et al., 2022; Yang et al., 2023). The role of social and environmental factors as either mediators or moderators in these relationships is also underexplored (Czerwiński and Atroszko, 2020). Additionally, the adaptation and validation of assessment instruments in the Chinese cultural and occupational context remain an area requiring more scholarly focus. Overall, a more integrated approach that considers individual (Li Z. M. et al., 2020; Li T. et al., 2020; Li Y. et al., 2022), occupational, and cultural variables could offer a more comprehensive understanding of how personality traits affect QoL in Chinese subpopulations (Feher and Vernon, 2021).

### Limitations of the present scoping review

The present scoping review is subject to several limitations that must be acknowledged for a comprehensive understanding of its findings. First, the review is confined to studies conducted within the Chinese population, potentially limiting the generalizability of the findings to other cultural (Yang H. et al., 2022; Yang P. Y. et al., 2022) or ethnic groups (Meléndez et al., 2019). Second, the review primarily relies on studies employing self-report questionnaires for the assessment of Big Five personality traits and QoL. Self-report measures are susceptible to biases, including social desirability, that may impact the validity of the results.

Furthermore, the studies included in the review exhibit a range of methodological designs and quality, with many relying on cross-sectional data. The cross-sectional nature of these studies precludes causal inferences about the relationships between personality traits and QoL. Longitudinal studies are needed to confirm the directionality of these relationships. Additionally, the review observes a lack of uniformity in the instruments used across the studies, making it challenging to compare results directly or conduct a meta-analysis.

Another limitation is the focus on specific subpopulations, such as judges, civil servants, and people with disabilities, which may not be representative of the broader Chinese population. Moreover, the mediating factors like social support, occupational stress, and resilience are not consistently examined across all studies, which hampers the ability to synthesize findings effectively (Robinson et al., 2010).

Lastly, the review does not provide an exhaustive account of all potential mediating or moderating factors that could influence the relationship between Big Five personality traits and QoL. Factors like socioeconomic status, education level, and specific health conditions are sporadically addressed in the reviewed studies but require more systematic investigation (Silva et al., 2022).

Despite these limitations, the review offers valuable insights into the complex relationships between Big Five personality traits and QoL within the context of Chinese subpopulations. Future research should aim to address these limitations to provide a more comprehensive and nuanced understanding of these relationships.

## Conclusion

In conclusion, this scoping review critically examines the relationship between the Big Five personality traits and Quality of Life (QoL) in China’s evolving cultural and socio-economic landscape. Our systematic analysis, employing the PRISMA-ScR framework and “C-BFQLC” protocol, reviewed 170 documents, with nine meeting our stringent criteria. These studies illuminate the complex interaction between personality traits and QoL, highlighting the need for more in-depth research in this nascent area. Our findings have significant implications for healthcare, organizational behavior, and social welfare in China, particularly for subgroups like the elderly and those with disabilities. This research contributes to understanding the role of personality in QoL and underscores the importance of cultural considerations in well-being, laying the groundwork for future research and targeted interventions in China.

## Data availability statement

The original contributions presented in the study are included in the article/supplementary material, further inquiries can be directed to the corresponding author.

## Author contributions

CC: Formal analysis, Methodology, Software, Visualization, Writing – original draft, Writing – review & editing.
